# 

**Published:** 1844-09

**Authors:** 


### CONTENTS

OF NO. IX.

---

### ORIGINAL COMMUNICATIONS.

### ESSAYS AND CASES.

Art. I.—Epidemic Typhus Fever in Ohio. By Jno. Dawson. M.D., of Jamestown, Ohio. - - - - 185

Art. II.—Surgical Cases. By Wm. M. Boling, M.D., of Montgomery, Alabama. - - - - - - 218

### REVIEWS.

Art. III.—A Demonstration of the Curability of Pulmonary Consumption in all its Stages. Comprising an Inquiry into the Nature, Causes, Symptoms, Treatment, and Prevention of Tuberculous Diseases in general. By Wm. A. M'Dowell, M. D. Louisville, Ky. Prentice & Weissinger. 1843. pp. 269. 8vo - 242

Art. IV.—The Hospitals and Surgeons of Paris. An Historical and Statistical account of the Civil Hospitals of Paris, with Miscellaneous Information, and Biographical Notices of some of the most eminent of the living Parisian Surgeons. By F. Campbell Stewart, M.D. N. York: J. & H. G. Langley. Philadelphia: Carey & Hart. pp. 432. 8vo. 1843. - 248

SELECTIONS FROM AMERICAN AND FOREIGN JOURNALS.

Enormous Tumor developed in the substance of the Womb - 257
Belladonna in Scarlatina. - - - - - - - 261
Case of Spontaneous Removal of a Cataract. - - - 262
Alcoholic Lotion in Phthisis Pulmonalis. - - - 263
Liberia—its Fevers—Longevity of its Inhabitants. - - 265
Physiological Effects of the Iodide of Potassium. - - 267
Sir Henry Halford. - - - - - - - - - 269

ORIGINAL INTELLIGENCE.

Traveling Letters, No. IV. - - - - - - - 270
The Western Lancet. - - - - - - - - 279
Medical Lectures. - - - - - - - - - 280
Books Received. - - - - - - - - - 280

